# Five-Year Clinical Performance of Complex Class II Resin Composite and Amalgam Restorations—A Retrospective Study

**DOI:** 10.3390/dj11040088

**Published:** 2023-03-24

**Authors:** Maria Jacinta M. C. Santos, Heleine Maria C. Rêgo, Imad Siddique, Abbas Jessani

**Affiliations:** 1 Division of Restorative Dentistry, Schulich School of Medicine and Dentistry, The University of Western Ontario, Room # 0149, Dental Sciences Building, London, ON N6A 5C1, Canada; 2 Department of Epidemiology and Biostatistics, Schulich School of Medicine and Dentistry, The University of Western Ontario, London, ON N6A 5C1, Canada

**Keywords:** amalgam, clinical evaluation, complex class II restoration, resin composite, restorative dentistry

## Abstract

The aim of this retrospective study was to investigate the clinical performance of posterior complex resin composite (RC) and amalgam (AM) restorations after a five-year period. One hundred and nineteen complex Class II restorations placed by dental students were evaluated using the USPHS criteria. Data were analyzed using Chi-square, Mann–Whitney, and Wilcoxon tests at a 0.05 level of significance. After five years, the percentages of clinically satisfactory complex Class II RC and AM restorations were 78% and 76.8%, respectively. The main reasons for the failure of AM restorations included secondary caries (Bravo—10.1%), defective marginal adaptation (Charlie—8.7%), and fracture of the tooth (Bravo—7.2%). RC restorations presented failures related to the fracture of the restoration (Bravo—16%) and defective marginal adaptation (Charlie—8.2%). There was a significantly higher incidence of secondary caries for AM restorations (AM—10.1%; RC—0%; *p* = 0.0415) and a higher number of fractures for RC restorations (AM—4.3%; RC—16%; *p* = 0.05). Regarding anatomy, AM restorations presented a significantly higher number of Alfa scores (49.3%) compared to RC restorations (22.4%) (*p* = 0.0005). The results of the current study indicate that complex class II RC and AM restorations show a similar five year clinical performance.

## 1. Introduction

Among direct restorative materials, amalgam has been utilized for almost 200 years. Historically, it has been shown to restore complex Class II restorations in posterior teeth due to its high compressive strength, low wear rate, low cost, and long-term survival [1]. Over the past two decades, a continuous shift toward the use of resin composite materials has been observed [2,3,4], and the use of amalgam has declined due to its unaesthetic appearance and environmental concerns related to the presence of mercury [5,6,7]. In 2013, the Minamata Convention on Mercury proposed a gradual phase-down of amalgam to slowly eliminate the use of mercury-containing products [8]. Its main aim was to eliminate the main sources of mercury pollution, including dental amalgams [9,10]. The convention was ratified in 2017 and supported ongoing research into the development of alternative restorative materials to amalgam. Parallel to the concerns raised about mercury pollution, there has been an ongoing trend toward the use of resin composite restorations in extensive cavity preparations [7,11]. The cure-on command allied with the visco-elastic properties of the resin composite material facilitates placement, shaping, and contouring when replacing dental structures [12]. Moreover, the use of adhesive materials promotes a strengthening effect on the remaining tooth structure due to its micromechanical bond to dental tissues [13].

The use of resin composite materials to restore posterior teeth in dental schools became more popular in the late 1990s, and since then, increased teaching time has been dedicated to this material [14,15,16]. Although dental schools in Canada [2], the United States [3], Europe [17], and other parts of the world [15] continue teaching amalgam as part of their curriculum, some recent studies have emphasized the need to increase the time dedicated to teaching resin composite restorations in complex cavity preparations to reflect the increased use of resin composites in the clinical setting [2,3]. To date, there is still wide variation regarding the indications, contraindications, and techniques that are taught for posterior composite restorations in dental schools [2,14], despite substantial reports about the significant shift toward the use of this material in dental school clinics [2,14,15,16].

The main drawbacks of resin composite materials are related to their technique sensitivity and inherent polymerization shrinkage properties [18,19]. The use of an incorrect technique can result in postoperative sensitivity, microleakage, and excessive wear [7,16,20]. Moreover, the use of a resin composite in complex Class II posterior restorations with cusp replacement remains controversial. Insufficient data from long-term studies and the consequent lack of long-term clinical evidence restrain the indication of composites for cusp-cap restorations.

Earlier studies have shown similar or superior survival of the composite compared to amalgam in class II restorations [4,21,22,23,24,25], while others have reported the superior survival of amalgam restorations compared to resin composites [7,26,27,28,29,30]. However, due to the low reported annual failure rate of composite restorations [4,11,31,32,33] and the continuous improvement of resin-based materials, the comparison of the clinical effectiveness of the complex class II resin composite and amalgam restorations is warranted.

The most common clinical criteria used to evaluate restorations are the United States Public Health Service (USPHS) or “Ryge criteria” [34,35], and those of the World Dental Federation (FDI), introduced in 2007 [36]. Both sets of criteria evaluated restorations considering their biological, functional, and esthetic aspects. While the USPHS criteria adopted Alfa, Bravo, Charlie, and Delta codes to record different aspects of the restoration evaluated, the FDI criteria use five scores (1. clinically very good; 2. clinically good; 3. clinically sufficient/satisfactory, 4. clinically unsatisfactory—repairable restoration, and 5. clinically poor—restoration replacement) [37]. Both the USPHS and FDI criteria are easily reproducible and can be tailored to the user’s needs.

The main objective of this retrospective study was to evaluate the performance of the complex class II resin composite and amalgam restorations placed by dental students over a five-year period. The null hypothesis is that the clinical performances of the complex class II resin composite and amalgam restorations after a five-year evaluation are similar.

## 2. Materials and Methods

This retrospective study was conducted according to the research guidelines involving human subjects by the World Medical Association Declaration of Helsinki, and was independently reviewed and approved by the Research Ethics Board for Health Sciences Research Involving Human Subjects (HSREB) (# 109006) at Western University. Written informed consent was obtained before the clinical evaluation of each patient. The inclusion criteria included (1) patients who had a dental treatment conducted at the school’s dental clinic five years ago, and (2) had a complex amalgam (three or more surfaces) or resin composite restorations (three or more surfaces) placed on molar or premolar areas by dental students, as identified through the school billing system. These patients were contacted by phone or email and were invited for a follow-up examination at the school’s dental clinic.

All restorations were placed by third and fourth-year dental students under the supervision of faculty members in the Department of Restorative Dentistry. The treatment decision between a complex direct restoration and an indirect partial or full coverage crown was based on several factors including the patient’s medical and dental histories, the number and clinical condition of the remaining walls, periodontal condition, pulp status, occlusion, the number of teeth present in the mouth, esthetic requirement, chair time, and cost.

The operative protocol for amalgam and resin composite restorations involved the following steps: local anesthesia; rubber dam isolation (or when its placement was not possible, cotton rolls, dry angles, and high-volume suction were used); cavity preparation; the use of pins and/or other retentive features when necessary; the use of calcium hydroxide (Dycal—Dentsply Sirona) and/or RMGI (Vitrebond—3M/ESPE) liners in deep cavity preparations of vital teeth; metal matrix placement and sycamore wooden wedges. The Tofflemire matrix and retainer were most frequently used with amalgam restorations, while the circumferential Automatrix (Dentsply Sirona) or sectional matrices with an elastic ring (Garrison Dental Solutions) were used with resin composite restorations. The additional steps for the resin composite restorations included acid conditioning of the dental tissues with 37% phosphoric acid for 15 s. (total-etch approach), followed by water rinsing and gentle air-drying. A dentin-bonding agent (Peak™ Universal Bond—Ultradent Products) was applied and light-cured for 10 s. The resin composite (Filtek Supreme-3M-ESPE) was placed incrementally and light-cured for 20 s using LED light-curing units (Bluephase Style, Ivoclar Vivadent). After occlusion adjustments, finishing and polishing were performed using fine diamond burs (Brasseler) and rubber points (Cosmedent). Additionally, manual instruments such as a gold knife (Brasseler) and Sof-lex discs (3M-ESPE) were used to remove any excess on facial, lingual, and occlusal embrasures. Amalgam restorations were performed with the non-gamma 2 admix alloy (Sdi Permite Amalgam Capsules) and were polished at least 48 h after placement using Dura Green pointed stones (Shofu, Inc.- 8-1, Aketa-cho, Takatsuki-shi, Osaka 569-1147, Japan) and rubber points (Shofu, Inc.). The main restorative materials that were used in this study are listed in Table 1.

During the follow-up examination at the dental school clinic, patients were informed about the research methodology, risks, and benefits of their one-time participation in this study. A full understanding and written informed consent were obtained before the restorations were assessed by two independent investigators (HR, JS) calibrated in the use of the system using the modified USPHS criteria by rating the complex resin composite and amalgam restorations during triage appointments at the dental school clinic. [34] (Table 2). The investigators were calibrated by the PI and independently evaluated the restorations using mirrors and probes. In the presence of any disagreement, a re-evaluation of the restorations was performed, and a consensus was achieved before the rating. The intra-examiner Cohen’s kappa was 0.85. The patients were asked about their satisfaction with the restorations and about the presence of sensitivity or discomfort after the placement of the restorations or during mastication. If any restoration needed repair or replacement, patients were advised to contact the dental clinic to book an appointment for treatment. Clinical photographs were taken of select patients for illustration. The inter-examiner reliability was determined to be above 0.92 for all criteria, demonstrating a high rate of agreement between the examiners. The date of placement and the date of the last observation of restoration were recorded for statistical analysis. Data were statistically analyzed using a Chi-squared test to determine whether there was a relationship between each feature and type of restoration, followed by Mann–Whitney and Wilcoxon tests.

## 3. Results

Among the 125 patients contacted, 45 did not participate in this study for various reasons. A total of eighty (80) patients, including males (n = 42) and females (n = 38) with ages ranging from 26–86 (mean age = 65.5) were evaluated. A total of 69 amalgams (12 pre-molars, 57 molars) and 50 resin composite restorations (30 pre-molars, 20 molars) were evaluated. Each patient’s caries risk was assessed, and restorations were evaluated according to the modified USPHS criteria for postoperative sensitivity, secondary caries, marginal adaptation, the fracture of the restoration, the fracture of the tooth, and anatomy. These aspects of the criteria were scored as A (Alfa), B (Bravo), C (Charlie), or D (Delta) (Table 2).

Table 3 summarizes the results obtained in the clinical evaluation of the restorations at the recall appointment five years after placement. Among the 119 restorations assessed, 76.8% of the amalgam and 78.0% of the resin composite restorations were considered satisfactory. There was a higher incidence of secondary caries in amalgam restorations compared to resin composite restorations at the five-year evaluation (*p* = 0.0415). Conversely, a higher incidence of restoration fracture (*p* = 0.05) and poor anatomy (*p* = 0.0005) was identified in composite restorations. Despite the different reasons for failure, no statistically significant difference was found between the survival rate of the two restorative materials (*p* = 0.879) (Table 4).

**Figure 1:**
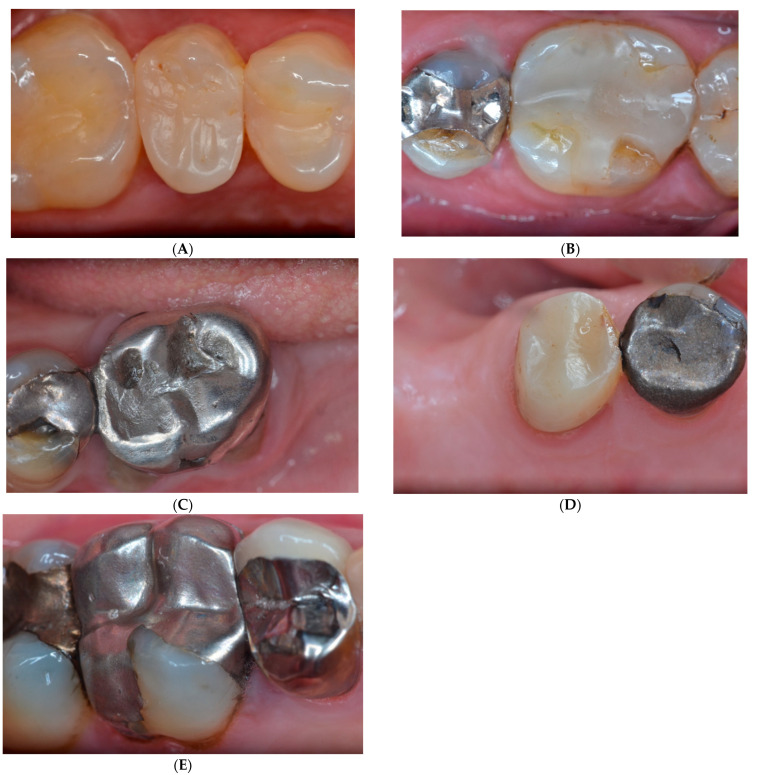
Photos (**A**–**E**) illustrate resin composite and amalgam complex restorations evaluated in the present study. (**A**)-MODL composite restoration in the second upper premolar; (**B**)-MODBL composite restoration in the first lower molar; (**C**)-MODBL amalgam restoration in the first lower molar; (**D**)-MODBL composite restoration in the second lower premolar; (**E**)-MODBL amalgam restoration in the first upper molar.

The major reasons for the failure of the amalgam restorations were secondary caries (Bravo—10.1%), defective marginal adaptation (Charlie—8.7%), and the fracture of the tooth (Bravo—7.2%). Most of the failures with resin composite restorations were due to the fracture of the restoration (Bravo—16%) and defective marginal adaptation (Charlie—8.2%). There were significant differences between the two restorative materials for secondary caries (AM—10.1%; RC—0%), the fracture of the restoration (AM—4.3%; RC—16%), and anatomy, in which AM presented a higher number of Alfa scores (49.3%) compared to RC restorations (22.4%) (Table 3). However, no difference was found in the survival of the complex class II amalgam and resin composite restorations. Figure 1A–E illustrates some restorations that were evaluated in the present study.

## 4. Discussion

The aim of this study was to verify the current performance of resin composite restorations in complex cavity preparations compared to amalgam (most investigated in complex preparation designs), as the latter material is being phased out. The number of surfaces involved in the cavity preparation is considered the most relevant aspect influencing the clinical success of posterior direct restorations [23,24,26,27]. Studies have shown that complex class II restorations are more prone to clinical complications, resulting in shorter longevity [23,26,30]. Opdam and colleagues [30] observed that large restorations presented a higher risk of failure and stated that every extra surface included in a restoration increased this risk by 30–40%. The main reported failures of complex class II direct restorations were related to the fracture of the restoration, secondary caries, and marginal discrepancies [7,23,24,26]. The clinical performance and longevity of amalgam and resin composite restorations have been evaluated in several clinical trials. However, the results are still controversial. While some studies reported more favorable results for amalgam compared to resin composite restorations [7,26,27,28,29,30], other studies described similar performances between the two restorative materials, as identified in our study [4,21,22,23,24].

The results of the present study showed comparable clinical performances of complex class II amalgam and resin composite restorations (76.8% and 78%, respectively), with no significant differences between the two materials. These findings are in agreement with several previous studies [4,21,22,23,24]. Mannocci (2005) [21], after a five-year prospective evaluation period, reported a survival rate of 90% and 91.3% of premolars restored with amalgam and resin composites, respectively. Palotie and colleagues (2017) [24], in a retrospective study, reported a similar longevity of complex class II composite and amalgam restorations, and stated that the survival rate of three-surface restorations was challenging for both materials. In contrast to these results, Opdam and colleagues observed a better survival rate of large posterior composite restorations compared to large amalgam restorations after a 12-year evaluation, especially in the low caries-risk patients. [25], while other studies have reported the superior performance of amalgam restorations compared to resin composites [7,26,27,28,29,30]. Several systematic reviews [38,39,40] reported the superior clinical performance of amalgam compared to resin composite restorations. However, systematic reviews that solely evaluated the clinical performance of resin composite restorations reported the clinical adequacy of this restorative material [11,22,40,41]. Beck and colleagues (2015) [11] reported that resin composite restorations presented an annual failure rate of 1.46% for short-term studies (1–4 years) and 1.97% for long-term studies (five years or more).

In the present study, secondary caries were the most significant factor attributed to amalgam failure, while the fracture of the restorative material was the main factor contributing to composite failure. The results observed in the present study are in agreement with previous reports [11,42]. Van Nieuwenhuysen and colleagues [28] evaluated complex class II restorations in posterior teeth and observed a higher incidence of secondary caries in amalgam restorations compared to the resin composite. Qvist et al. [42] reported that secondary caries was the most common reason for the replacement of failed amalgam restorations in permanent dentition. Additionally, it is relevant to consider that the use of amalgam is preferred in patients with high caries risk due to the material’s antibacterial properties, which are attributed to the presence and release of metallic ions such as silver, copper, and tin [43]. However, the use of amalgam on high caries-risk patients may corroborate a higher incidence of secondary caries on amalgam restorations in the present and previous studies [28,29,31,42]. Noaman et al. [44] reported a direct correlation between caries risk and oral hygiene, verifying an increased risk for the development of secondary caries and restoration replacement in high caries-risk patients, particularly for CI II restorations. Kim et al. [45] evaluated the impact of dental flossing in the adult population and verified that non-flossers presented approximately 1.5 times higher risk for proximal caries compared to flossers, since flossing on the teeth’s proximal surfaces physically reduces bacterial adhesion and cariogenic bacteria [45]. Regarding the failure of resin composite restorations, the report from a systematic review considered the fracture of the resin composite material as the main cause of restoration failure by up to five years, while the occurrence of secondary caries was observed only after five years of evaluation [11]. The low incidence of secondary caries during the first years of evaluation of resin composite restorations has been reported by several investigations [7,11,27,46]. Beck et al. [11] also reported that the fracture of resin composite restorations was the most common reason for failure in follow-up evaluations between one and four years, while the presence of secondary caries was observed after this period. Demarco et al. [46] confirmed the absence of secondary caries on resin composite restorations upon initial follow-up, and reported the presence of caries on recall evaluations between five and seven years. Moreover, previous studies have reported the fracture of the restoration as the most common reason for the failure of resin composite restorations [7,26,27,28,30]. Sookhakiyan and colleagues [47] stated that although the properties of resin composite materials have significantly improved to permit their use in the posterior load-bearing areas of the mouth, the fracture toughness of composites is compromised by water sorption. Unfortunately, the water degradation of restorative materials is hard to avoid, since they are constantly exposed to saliva and beverages in the oral environment.

For the amalgam restorations evaluated in the present study, the incidence of tooth fracture was one of the three most common causes of failure. Similar results were reported by other studies [23,27,28,29]. Kooperud and colleagues (2012) [27] considered that the fracture of the remaining tooth structure was the second major cause of failure after a 4.6-year evaluation. Opdam and colleagues [25] found that fracture and “cracked-tooth syndrome” were more prominent in amalgam than on resin composite restorations, and attributed the lower fracture rate of the teeth restored with a composite to the strengthening effected promoted by the adhesive technique. They reported a high incidence of tooth fracture and cracked-tooth symptoms on amalgam-restored teeth. Similarly, Naghipur and colleagues (2016) [29] reported that tooth fracture and secondary caries were the most common reason for amalgam restorations’ failure over a 12-year evaluation period.

Among all the aspects evaluated, marginal adaptation was the category that received the lowest number of Alfa scores for both amalgam and resin composite restorations. Marginal adaptation is relevant to the longevity of restorations. The presence of marginal gaps or defective margins may favor microinfiltration and result in secondary caries [12]. Estay and colleagues [48] reported that the main reasons for the repair and replacement of amalgam and resin composite restorations were related to the presence of defective margins and secondary caries. Duncalf and colleagues [49] evaluated the marginal adaptation of amalgam and resin composite class II restorations and observed a significantly higher number of marginal defects in the cervical segments of amalgam restorations, which presented a greater percentage of fissures and underfilled margins in most areas of the boxes compared to the composites. Regarding composite restorations, the main reasons for faulty marginal adaptation have been attributed to the intrinsic polymerization shrinkage of the restorative material, the long-term degradation of adhesive bonding [50], and cumulative fatigue under constant occlusal forces [51,52]. The polymerization contraction is inherent to composite materials. It happens due to the linking of monomers to form polymer chains of three-dimensional networks during the polymerization process, which leads to volume shrinkage in the form of intermolecular distance changes from 0.3–0.4 nm to 0.15 nm [19]. The stress of polymerization contraction can lead to cuspal flexure, enamel micro-cracking, bond failure, and microinfiltration, which can result in restoration failure [53]. In addition, other factors such as cavity configuration and restorative techniques may also influence composite adaptation [54]. Dačić and colleagues [55] evaluated the marginal gap of resin composite restorations performed with the etch-and-rinse technique and observed a significantly higher percentage of margins without gaps in the enamel (92.5%) compared to dentin (57.3%). This is an important fact to be considered, as a significant number of class II cavity preparations present the gingival margins located below the CEJ due to the gingival extension of carious tissues.

With reference to the other two aspects evaluated, restorations from both materials represented 97% of Alfa scores for post-operative sensitivity. Regarding anatomy, the amalgam restorations exhibited significantly better anatomy compared to the resin composite restorations. This finding is in agreement with a 12-year controlled clinical trial study that evaluated the effect of refurbishing amalgam and resin composite restorations, and verified a superior marginal adaptation for resin composite restorations and better anatomy for amalgam restorations.

A similar clinical performance between amalgam and resin composite complex class II restorations verified in the present study may be related to the improvement of the resin composite materials, which resulted in superior physical and mechanical properties, reduced volumetric shrinkage, increased wear resistance, and better capabilities for polishing [12,46]. Da Rosa and colleagues [31] verified superior longevity for the higher filler-loaded composite (mid-filled) with a constant annual failure rate between 10 and 20 years of evaluation. A recent systematic review [56] reported similar clinical behavior for complex class II amalgam and resin composite restorations. In their review, they verified the superior performance of resin composite restorations compared to amalgam in retrospective studies, while the opposite result was found in prospective studies. They related these findings to the increased probability of prospective studies investigating materials that are no longer available on the market. Over the last few decades, advancements in filler technology have resulted in the introduction of micro-hybrid and nanohybrid composites with a higher filler content based on the use of glass, zirconium, and silica [4,12,18,57]. Moraschini and colleagues (2015) [57] highlighted the importance of performing new clinical evaluations from time to time due to the constant improvements in resin-based materials and adhesive systems.

Regarding the evaluation criteria, some studies have considered FDI to be more sensitive to small variations in clinical outcomes than USPHS [58,59]. In contrast, other studies have observed no difference, and stated that both criteria worked equally well. [60,61] Although there has been a tendency toward using FDI criteria, the present study selected the USPHS criteria to allow for further comparison with previous studies, since several clinical trials continued to use USPHS criteria.

The limitation of this and other retrospective studies is the lack of baseline data for comparison with follow-ups. Moreover, because the patients’ charts were not initially designed to collect data for research, some detailed information may be missing. However, prospective studies require the long-term commitment of patients for recall visits, which can become challenging over time and may jeopardize the results of long-term evaluations [22]. Furthermore, randomized controlled trials (RCTs) use a controlled population, since many patients are excluded because they do not fit the inclusion criteria, which may not represent reality. The practice-based and retrospective design used in the present study allows for a better correlation with real case scenarios, and care was taken to utilize all relevant information from the patient’s charts. Regardless of the limitations, this is the first study of its kind to investigate the clinical performance of complex amalgam vs. composite restorations in a dental school setting in Ontario, Canada.

## 5. Conclusions

There was a higher incidence of secondary caries in complex class II amalgam restorations compared to resin composite restorations at a five-year post-operative evaluation (*p* = 0.0415). Conversely, a higher incidence of restoration fracture (*p* = 0.05) and poor anatomy (*p* = 0.0005) was identified in complex class II composite restorations. There was no significant difference in the survival rate of complex class II amalgam and resin composite restorations at the five-year evaluation (76.8% and 78%, respectively).

## Figures and Tables

**Table 1:** List of the main restorative materials used in this study.

Material	Brand	Composition	Manufacturer
Amalgam	SDI Permite	Ag 56%, Sn 27.9%, Cu 15.4%, In 0.5%, Zn 0.2%, Hg 47.9%	SDI, Bayswater, Victoria, Australia
RMGI Liner	Fuji II LC	HEMA, 25–50% polybasic carboxylic acid, 5–10% UDMA, plus trade secret components	GC, Fuji, Tokyo, Japan
Resin composite	Filtek Supreme	Bis-GMA, UDMA, TEGMA, bis-EMA, zirconia filler, silica filler	3M ESPE, St. Paul, MN, USA
Adhesive system	Peak Universal	Fluid resin with 7.5% filled, chlorhexidine (0.2%) plus trade secret components	Ultradent, South Jordan, UT, USA

HEMA (2-hydroxyethyl methacrylate), UDMA (urethane dimethacrylate), TEGMA (tri-ethylene glycol dimethacrylate), bis-EMA (2,2-bis (4-(2-Methacryl oxyethoxy) phenyl) propane).

**Table 2:** Modified USPHS (United States Public Health Systems) criteria used for clinical evaluation of restorations.

Category	Score	Criteria
Postoperative sensitivity	Alfa	No patient-reported sensitivity on the restored tooth
	Bravo	Patient-reported sensitivity on the restored tooth
Secondary caries	Alfa	No visual evidence of dark, deep discoloration adjacent to the restoration
	Bravo	Visual evidence of dark, deep discoloration adjacent to the restoration
Marginal adaptation	Alfa	Restoration closely adapted to the tooth. No crevice visible. No explorer-catch at the margins or there was a catch in one direction
	Bravo	Explorer-catch. No visible evidence of a crevice into which the explorer could penetrate. No dentin or base visible
	Charlie	Explorer penetrates into a crevice that is of a depth that exposes dentin or base
Fracture restoration	Alfa	No evidence of fracture
	Bravo	Evidence of fracture.
Fracture tooth	Alfa	No evidence of fracture
	Bravo	Evidence of fracture
Anatomy	Alfa	Restorations continuous with existing anatomic form
	Bravo Charlie	Restorations discontinuous with existing anatomic form but missing material not sufficient to expose dentin base material lost to expose dentin or base

**Table 3:** Amalgam and resin composite restoration scores according to the USPHS criteria.

Category	Score	Amalgam	Resin Composite	*p* Value
Postoperative sensitivity	Alfa	64 (97%)	45 (97.8%)	0.7756
	Bravo	2 (3%)	1 (2.2%)	
Secondary caries	Alfa	62 (89.9%)	50 (100%)	* 0.0415
	Bravo	7 (10.1%)	0 (0%)	
Marginal adaptation	Alfa	18 (26.1%)	11 (22.4%)	
	Bravo	45 (65.2%)	34 (69.4%)	0.7626
	Charlie	6 (8.7%)	4 (8.2%)	
Fracture of restoration	Alfa	66 (95.7%)	42 (84%)	* 0.05
	Bravo	3 (4.3%)	8 (16%)	
Fracture of tooth	Alfa	66 (92.8%)	49 (98%)	0.2354
	Bravo	5 (7.2%)	1 (2%)	
Anatomy	Alfa	34 (49.3%)	11 (22.4%)	* 0.0005
	Bravo Charlie	35 (50.7%) -	38 (77.6%) -	

* *p* value significant < 0.05.

**Table 4:** Failure rate comparison between amalgam and resin composite restorations.

	Amalgam	Resin Composite	*p* Value
No failure	53 (76.8%)	39 (78.0%)	0.879
Failure	16 (23.2%)	11 (22.0%)	
Total restorations	69 (100%)	50 (100%)	

*p* value significant < 0.05.

## Data Availability

Data are available on reasonable request to the corresponding author.

## Institution Board Review Statement

This study was conducted according to the research guidelines involving human subjects by the World Medical Association Declaration of Helsinki and was independently reviewed and approved by the Research Ethics Board for Health Sciences Research Involving Human Subjects (HSREB) (# 109006; date of approval: 29 March 2017) at the Western University.
